# Cold Physical Plasma-Mediated Fenretinide Prodrug Activation Confers Additive Cytotoxicity in Epithelial Cells

**DOI:** 10.3390/antiox12061271

**Published:** 2023-06-14

**Authors:** Mohsen Ahmadi, Debora Singer, Felix Potlitz, Zahra Nasri, Thomas von Woedtke, Andreas Link, Sander Bekeschus, Kristian Wende

**Affiliations:** 1 ZIK *plasmatis*, Leibniz Institute for Plasma Science and Technology (INP), Felix Hausdorff-Str. 2, 17489 Greifswald, Germany; 2 Clinic and Policlinic for Dermatology and Venereology, Rostock University Medical Center, Strempelstr. 13, 18057 Rostock, Germany; 3 Institute of Pharmacy, Greifswald University, Friedrich-Ludwig-Jahn-Str. 17, 17489 Greifswald, Germany; 4 Institute for Hygiene and Environmental Medicine, Greifswald University Medical Center, Sauerbruchstr., 17475 Greifswald, Germany

**Keywords:** boronic pinacol ester, cancer therapy, cold atmospheric pressure plasma, gas plasma technology, prodrug, reactive oxygen species (ROS)

## Abstract

Cold physical plasma is a partially ionized gas operated at body temperature and utilized for heat-sensitive technical and medical purposes. Physical plasma is a multi-component system consisting of, e.g., reactive species, ions and electrons, electric fields, and UV light. Therefore, cold plasma technology is an interesting tool for introducing biomolecule oxidative modifications. This concept can be extended to anticancer drugs, including prodrugs, which could be activated in situ to enhance local anticancer effects. To this end, we performed a proof-of-concept study on the oxidative prodrug activation of a tailor-made boronic pinacol ester fenretinide treated with the atmospheric pressure argon plasma jet kINPen operated with either argon, argon–hydrogen, or argon–oxygen feed gas. Fenretinide release from the prodrug was triggered via Baeyer–Villiger-type oxidation of the boron–carbon bond based on hydrogen peroxide and peroxynitrite, which were generated by plasma processes and chemical addition using mass spectrometry. Fenretinide activation led to additive cytotoxic effects in three epithelial cell lines in vitro compared to the effects of cold plasma treatment alone regarding metabolic activity reduction and an increase in terminal cell death, suggesting that cold physical plasma-mediated prodrug activation is a new direction for combination cancer treatment studies.

## 1. Introduction

**Figure 1:**
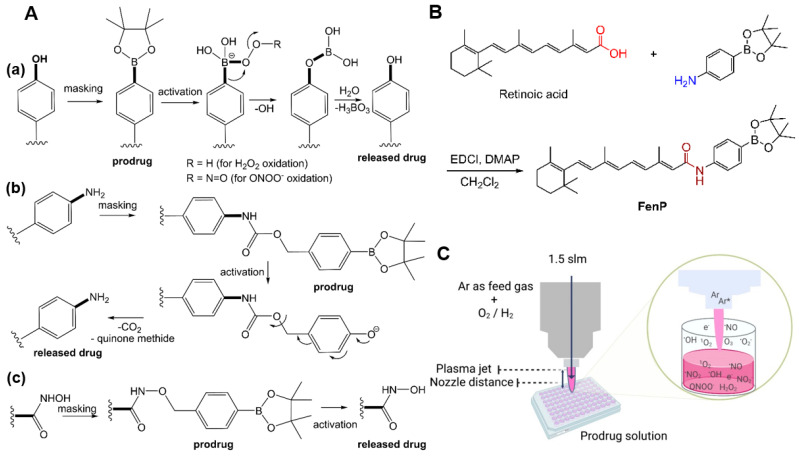
(**A**) Activation mechanism of arylboronic acid and esters prodrugs with (**a**) a phenol-based prodrug, (**b**) an aromatic nitrogen mustard prodrug, and (**c**) a belinostat prodrug; (**B**) synthesis of fenretinide prodrug; (**C**) schematic view of the cold-plasma-treated prodrug solutions and plausible reactive species production in the gas phase and liquid media. EDC–HCl = 1-ethyl-3-(3-dimethylaminopropyl)carbodiimide hydrochloride; DMAP = 4-dimethylaminopyridine; Ar = argon.

Various prodrug types and their corresponding activation mechanisms have been reported, e.g., overexpressed enzymes, pH changes, UV/NIR irradiation, activation by electromagnetic fields, photo-induced activation, and elevated reactive oxygen and nitrogen species (RONS) levels [1,2]. RONS-responsive prodrugs are currently receiving attention as efficient drug delivery tools [3,4]. These prodrugs often possess active functional groups, such as –OH, −NH_2_ [5], or –CONHOH [6], masked by a protective group that is eventually cleaved off at the target site [7]. Among others, (aryl)boronic acids and esters have been widely used as non-toxic RONS-sensitive protective groups [8], which are released upon exposure to RONS found in the tumor microenvironment. In the activation mechanism, the para-boronate group undergoes a 1,2 rearrangement, forming a phenyl-borate intermediate in a 1,2 rearrangement, which consequently turns into the corresponding phenol [9] (Figure 1A(a)). In addition, some prodrugs, for instance, the aromatic nitrogen mustard prodrug [5] and belinostat prodrug [6], undergo a 1,6-elimination reaction that involves the cleavage of a quinone methide moiety and the release of the final parent drug (Figure 1A(b,c)). Alternatively, RONS can be produced via photo-redox reactions, generating, e.g., hydrogen peroxide (H_2_O_2_), peroxynitrite (ONOO^−^), and hypochlorite (ClO^−^) [10,11]. Moreover, the stability of (aryl)boronic acid and ester-based prodrugs can be controlled, depending on the parent drug structure, pH, temperature, and concentration in the solution [12,13]. Furthermore, the field of cancer research has continued to witness the synthesis of novel molecules with potent anticancer properties in recent years [14,15,16,17].

Cold physical plasma (here, abbreviated as cold plasma) was originally developed for medical care. Today, this gas plasma technology has additionally become a toolbox for biomedical research in redox chemistry and biology by generating a variety of short- and long-lived reactive species, such as hydroxyl radicals (^•^OH), singlet oxygen (^1^O_2_), atomic oxygen (O(^3^P)), ozone (O_3_), nitric oxides (N_x_O_y_), and H_2_O_2_, which are capable of reacting with (bio-)molecules [18,19,20,21]. Besides the routine clinical application of medical gas plasma, such as the kINPen plasma jet, for wound healing in dermatology [22], oncological treatment regimes have been increasingly discussed [23]. Regarding the kINPen, we recently revealed anticancer effects against epithelial cells in vitro and in vivo, such as skin cancer cells [24,25]. In addition, we recently reported on a RONS-responsive boronate-based fluorocytosine prodrug that was activated by cold plasma in a time- and space-resolved manner [11]. The synthetic vitamin A analog fenretinide (N-4-hydroxyphenyl-retinamide) was used in this work. It possesses a highly reactive phenol moiety that can be eliminated by cellular metabolism. Fenretinide has shown cytotoxic activity against cancer cells in vitro [26,27,28], interfering with various cellular signaling pathways, including TRAIL and WNT [29,30,31,32]. Successful reports on its combinational cytotoxic effects with other anticancer drugs in vitro and in vivo further emphasize its suitability as a model compound [33,34,35]. However, the clinical application of fenretinide suffers from pharmacodynamic and pharmacokinetic issues (i.e., poor aqueous solubility (high lipophilicity), low selectivity, and high reactivity, necessitating improvement strategies, such as structural modifications or delivery via encapsulation or PEGylation, and combination therapies in order to increase the effectiveness and reduce dosing requirements) [9,36,37]. In this study, the active hydroxyl group of fenretinide was masked with a RONS-responsive boronate moiety in the first step (Figure 1B). In the second step, the free drug release by cold-plasma-generated RONS was investigated alongside the optimization of the plasma treatment conditions (Figure 1C). Thirdly, the impacts of the drug or prodrug, the cold plasma, and the combination thereof were tested in three epithelial cell lines in vitro. We hope that our study aids in further unraveling the relations between RONS, prodrugs, and potential therapies.

## 2. Materials and Methods

### 2.1. Materials

All starting materials, reagents, and solvents were commercially available and purchased from VWR, Abcr, or Carl Roth. Unless otherwise stated, the starting materials were used as provided. Thin-layer chromatography on an analytical scale was performed using silica gel 60 F_254_ aluminum plates supplied by Merck, and visualization was accomplished using UV light (254 nm). Flash column chromatography was carried out using an Interchim Puriflash XS 520Plus system and the appropriate 25 g silica gel cartridges available from Interchim (30-SI-HP). NMR analyses were run on a Bruker Biospin Avance III instrument at 400 MHz (^1^H) and 101 MHz (^13^C) using DMSO-*d*_6_ as the solvent. Chemical shifts are given relative to the internal standard tetramethylsilane and are reported in parts per million (ppm).

### 2.2. Prodrug Synthesis

Prodrug synthesis was conducted as previously reported in a patent [38]. Briefly, retinoic acid (500 mg, 1.66 mmol, 1.0 eq), 4-(4,4,5,5-tetramethyl-1,3,2-dioxaborolan-2-yl)aniline (365 mg, 1.66 mmol, 1.0 eq), EDCI (310 mg, 2.00 mmol, 1.2 eq), and DMAP (203 mg, 1.66 mmol, 1.0 eq) were dissolved in dichloromethane (20 mL). The vessel was flushed with argon gas, and the content was stirred at 20 °C for 16 h in the dark. Solvents were removed under reduced pressure, and the mixture was subjected to flash chromatography utilizing an *n*-hexane/ethyl acetate gradient. The title compound was isolated as a yellow oil, which slowly solidified on standing with a 22% yield. Experimental data follow the previously reported synthesis. *R*_f_ = 0.45 (10% ethyl acetate in *n*-hexane); ^1^H NMR (400 MHz, DMSO-*d*_6_): δ (ppm) = 10.11 (s, 1H, NH), 7.67 (d, *J* = 8.5 Hz, 2H, 2xC*H*_phenyl_), 7.60 (d, *J* = 8.5 Hz, 2H, 2xC*H*_phenyl_), 6.97–7.07 (m, 1H, C*H*_polyene_), 6.34–6.41 (m, 1H, C*H*_polyene_), 6.23–6.33 (m, 2H, 2xC*H*_polyene_), 6.14–6.22 (m, 1H, C*H*_polyene_), 6.05 (s, 1H, C*H*_polyene_), 2.36 (s, 3H, C*H*_3_), 1.95–2.05 (m, 5H, C*H*_2_ + C*H*_3_), 1.70 (s, 3H, C*H*_3_), 1.54–1.62 (m, 2H, C*H*_2_), 1.42–1.47 (m, 2H, C*H*_2_), 1.28 (s, 12H, 4xC*H*_3_), 1.02 (s, 6H, 2xC*H*_3_); ^13^C NMR (101 MHz, DMSO-*d*_6_): δ (ppm) = 12.6 (*C*H_3_), 13.3 (*C*H_3_), 18.7 (*C*H_2_), 21.5 (*C*H_3_), 24.7 (4x*C*H_3_), 28.8 (2x*C*H_3_), 32.6 (*C*H_2_), 33.8 (*C*), 39.2 (*C*H_2_), 83.4 (2x*C*), 118.0 (2x*C*H_phenyl_), 122.4 (*C*H_polyene_), 127.6 (*C*H_polyene_), 129.4 (*C*_polyene_), 130.1 (2x*C*H_polyene_), 135.2 (2x*C*H_phenyl_), 135.9 (*C*H_polyene_), 136.9 (*C*H_polyene_), 137.2 (*C*_phenyl_), 138.4 (*C*_phenyl_), 142.4 (*C*_polyene_), 148.9(*C*_polyene_), 164.9 (*C*=O). TripleTOF-MS (*m/z*), calculated for [C_32_H_44_BNO_3_ + H]^+^: 502.34; found: 502.37.

### 2.3. Sample Preparation

A 50 mM DMSO stock solution of the fenretinide prodrug (FenP) and parent drug (FenD) (Sigma-Aldrich, Taufkirchen, Germany) was prepared. The prodrug was diluted in MeOH (1:1, *v*/*v*) to a concentration of 500 μM and then diluted once with MeOH–H_2_O to a final concentration of 50 μM and incubated for 72 h to obtain the hydrolyzed compound (FenH) utilized for each treatment experiment in liquid. The evaporated amount of solvent during the cold plasma treatment was calculated and added to reach the same solvent volume before treatment. Treated samples were divided into aliquots, and 5 μM solutions of treated samples were subjected to Ultra-High-Performance Liquid Chromatography (UHPLC) with high-resolution mass spectrometry (TripleTOF MS^2^). The resulting degradation products were assessed from directly injected samples generated after being either left untreated, exposed to cold physical plasma for several treatment times (e.g., 5 s, 10 s, 20 s, 40 s, or 60 s), or exposed to chemically generated H_2_O_2_ at varying final concentrations. Standard calibration curves of FenD and FenH were prepared using five samples with concentrations of 0.78, 1.56, 3.12, 6.25, and 12 μM in solution.

### 2.4. Cold Physical Plasma Exposure

All experiments were performed at ambient temperature and atmospheric pressure using a kINPen IND (neoplas tools, Greifswald, Germany). The plasma jet consists of a grounded ring electrode enclosing a ceramic capillary, where a powered central rod electrode is located inside (2–6 kVpp at 1.1 MHz). The gas flow rate of argon (99.999% purity; Air Liquide, Bremen, Germany), serving as the main feed gas, was set at 1.5 standard liters per minute. In some experiments, 0.5% H_2_ or 0.5% O_2_ (Air Liquide, Bremen, Germany) was admixed as an extra feed gas. One hundred microliters of the diluted solution (50 μM) was placed in a 96-well flat-bottom plastic plate and treated with a plasma jet nozzle-to-liquid distance of 9 mm for each gas condition.

### 2.5. Mass Spectrometry (MS) Analyses

(U)HPLC-MS^2^ analysis was conducted on an Agilent 1290 Infinity LC system coupled to a QTRAP 5500 (AB Sciex, Darmstadt, Germany) as a Linear Ion Trap Quadrupole mass spectrometer. The capillary voltage was set to a value of 3.8 kV. The ion source temperature was set to 250 °C. The prodrug degradation products were separated using an HPLC system with EclipsePlus C-18 RRHD BEH (50 × 2.1 mm, 1.8 μm), with the corresponding pre-column. The column temperature was set at 30 °C. The mobile phase consisted of a buffer solution counting 15 mM ammonium formate (channel A) and acetonitrile (channel B). The separation was performed by gradient elution of 55% A for 30 s, 55% A for 1 min, 60% A for 30 s, 60% A for 6 min, 65% A for 30 s, 65% A for 1 min, 90% A for 30 s, 90% A for 3 min, 50% A for 30 s, and then 50% A for 30 s (total run time 14 min). The flow rate was set to 0.7 mL min^−1^, and the injection volume was 5 μL. Ionization was performed in positive mode. The full-spectrum ion transitions (multiple reaction monitoring: MRM), collision energies, and fragment energies were set as (i) *m/z* 502.3 → 283.2 (optimal collision energy (CE) = 20 eV) and 161.0 (CE = 30 eV) for FenP; (ii) *m/z* 420.2 → 283.2 (CE = 20 eV) and 161.0 (CE = 30 eV) for FenH; (iii) *m/z* 392.2 → 283.2 (CE = 20 eV) and 161.0 (CE = 30 eV) for FenD. For quantification, external calibration curves were generated. To elucidate the prodrug degradation products under cold plasma treatment and control experiments after the direct addition of H_2_O_2_, high-resolution mass spectrometry was performed using a TripleTOF 5600 system (AB Sciex, Darmstadt, Germany). Survey spectra acquisition was performed in a range of 20–1000 *m/z* in positive and negative modes via the direct infusion of diluted samples. All samples were injected using identical system settings (capillary temperature: 250 °C; curtain gas: 35 psi N_2_; ion source gas 25 psi N_2_; ion spray voltage: 3.8 kV). Each identified degradation product’s fragmentation spectrum (MS^2^) was acquired in product ion mode (CE: 10–70 eV, declustering potential: 80 V).

### 2.6. RONS Analysis

Hydrogen peroxide (H_2_O_2_) was quantified via its reaction with titanium(IV) oxysulfate (TiOSO_4_, 5% Ti(IV)) in sulfuric acid (Sigma-Aldrich, Taufkirchen, Germany) according to previously published protocols [39] and quantified at 407 nm with a spectrophotometer (Infinite M200 Pro; Tecan, Männedorf, Switzerland). Peroxynitrite (ONOO^−^) was assessed using the fluorescent dye hydroxyphenyl fluoresceine (HPF; Thermo Fisher, Dreieich, Germany) at *λ*_ex_ 485 nm and *λ*_em_ 525 nm with a spectrophotometer (Infinite F200 Pro; Tecan, Männedorf, Switzerland), as described before [40].

### 2.7. Cell Culture and Cold Plasma and Drug Treatment

The human epithelial squamous cell carcinoma cell lines A431 (skin; ATCC: CRL-1555) and SCaBER (urinary bladder; ATCC: HTB-3) and the non-malignant keratinocyte cell line HaCaT (skin; CLS: 300493) were used in this study. Cells were cultured in Roswell Park Memorial Medium (RPMI) 1640 supplemented with 10% fetal bovine serum, 2% L-glutamine, and 1% penicillin/streptomycin (all Sigma-Aldrich, Taufkirchen, Germany) under standard conditions (37 °C, 5% CO_2_, 95% humidity). The day before treatment, 5 × 10^3^ cells per well in 100 µL of fully supplemented medium were seeded in 96-well flat-bottom plates. The next day, the medium was exchanged for 90 µL of medium containing 4′,6-diamidino-2-phenylindole (DAPI, final concentration 1 µM; BioLegend, Amsterdam, The Netherlands). Besides those remaining untreated, the cells were treated with FenP or FenD (diluted in PBS; final concentration 20 µM) or exposed to cold physical plasma for 15 s, as described above. For combination treatments, two settings were applied: cold plasma treatment followed by drug addition (timing 1) or drug addition prior to plasma treatment (timing 2). Treatments with PBS served as vehicle controls.

### 2.8. Metabolic Activity and Viability Analysis

Metabolic activity was determined using the resazurin-based assay 72 h after cold plasma or drug exposure. Resazurin (final concentration: 100 µM) was added to the cells and incubated for 4 h. The capability of metabolically active cells to reduce resazurin to resorufin was quantified by measuring its mean fluorescence intensity (MFI) captured using a microplate reader (Infinite F200 pro; Tecan, Männedorf, Switzerland) at λ_ex_ 535 nm and λ_em_ 590 nm. The coefficient of drug interaction (CDI) was calculated as follows: CDI = AB/(AxB), with AB being the ratio of combination treatment to control MFI and A or B being the ratio of single-treatment (plasma or drug) to control MFI. For live-cell high-content imaging and viability assessment, images of the cells were acquired at 0 h (immediately prior to treatment), 24 h, 48 h, and 72 h post-treatment using a high-content imaging device (Operetta CLS; PerkinElmer, Hamburg, Germany) with a 10× (NA 0.3) objective (Zeiss, Jena, Germany). The channels used were brightfield (BF), digital phase contrast (DPC), and the λ_ex_ 365 nm and λ_em_ 465 ± 35 nm fluorescence channels to capture DAPI fluorescence of terminally dead cells. Images were analyzed using *Harmony* 4.9 (PerkinElmer, Hamburg, Germany) software.

### 2.9. Visualization and Data and Statistical Analysis

Data acquisition, peak integration, and calculation were performed using analyst, Peakview software (version 1.1.1.2; AB Sciex, Darmstadt, Germany), and Prism 9.5.1 (GraphPad Software, San Diego, CA, USA). Determination of IC_25_ (IC_50_) values was performed using nonlinear regression analysis against log2-transformed exposure times. All experiments were performed three times. Statistical analysis was performed using one-way analysis of variances (ANOVA) with Dunnett’s post hoc test against the vehicle control if not indicated otherwise in the figure legends. The significance level was α = 0.05, and statistical significance is indicated as follows: *p* ≤ 0.05 (*), *p* ≤ 0.01 (**), and *p* ≤ 0.001 (***).

## 3. Results and Discussion

### 3.1. Prodrug Synthesis

**Figure A1:**
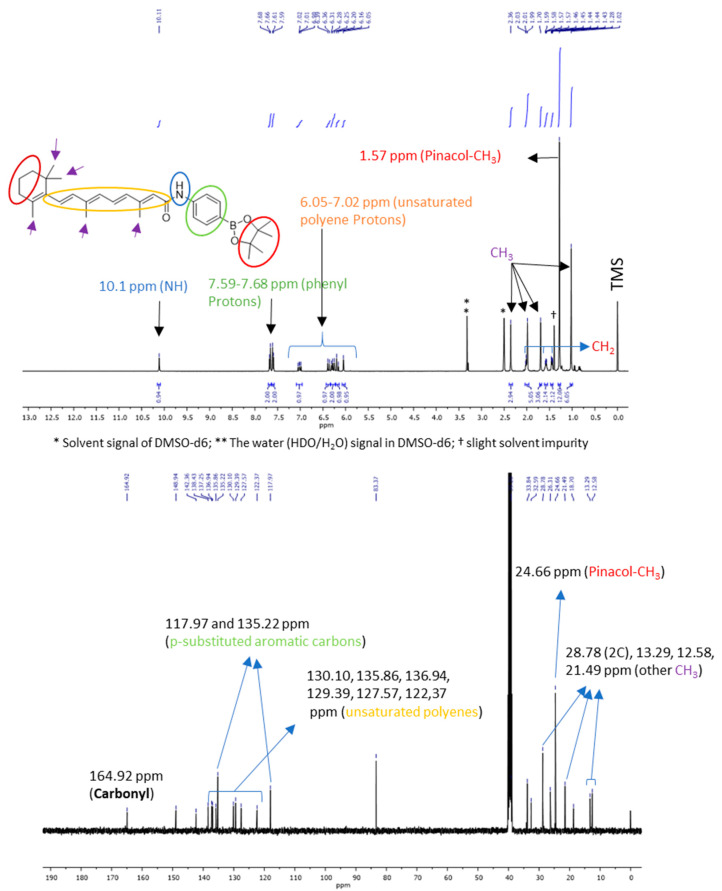
^1^H NMR (**top**) and ^13^C NMR (**bottom**) of prodrug (FenP, (2E,4E,6E,8E)-3,7-dimethyl-N-(4-(4,4,5,5-tetramethyl-1,3,2-dioxaborolan-2-yl)phenyl)-9-(2,6,6-trimethylcyclohex-1-en-1-yl)nona-2,4,6,8-tetraenamide).

**Figure A2:**
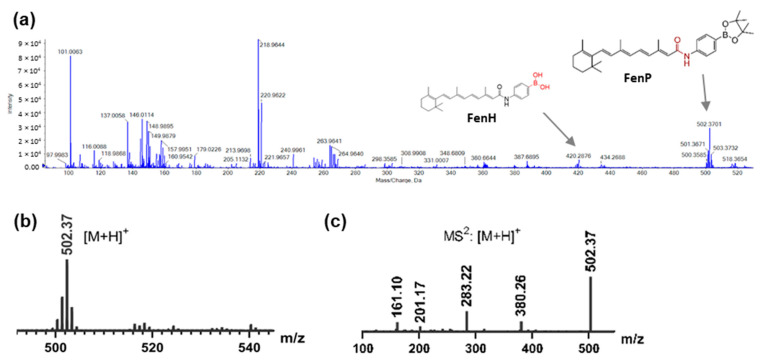
(**a**) ESI-MS(+), (**b**) expanded ESI-MS(+), and (**c**) ESI-tandem MS^2^ spectrum of fenretinide prodrug matching the [M + H]^+^.

The EDCI/DMAP-mediated amide coupling reaction is frequently utilized in medicinal chemistry to activate carboxylic acid groups for reactions with an amine [41]. The fenretinide prodrug was synthesized from retinoic acid in a one-step reaction procedure (Figure 1A). Product purification was performed via column chromatography. The ^1^H NMR spectrum of the isolated compound revealed a low-field and high-frequency shift to 10.1 ppm for the amide NH group and the vanishing of the NH_2_ protons of the aniline derivative, indicating the anticipated prodrug’s successful formation. A singlet at 1.28 ppm corresponding to twelve protons belonging to the two pinacol residues was also observed in ^1^H NMR, whereas the corresponding signal could be found at 24.7 ppm in the ^13^C NMR spectrum (Figure A1). Electrospray ionization spectrometry (ESI-MS(+)) showed a signal at *m/z* 502.37 (Figure A2(a,b)). Utilizing ESI-MS^2^ (TripleTOF 5600), analyses identified the fragmentation (daughter) products by changing the collision energy to 30 eV at *m/z* 283.22 for the amide bond cleavage (Figure A2(c)) belonging to retinal.

### 3.2. Cold Plasma-Generated RONS

**Figure 2:**
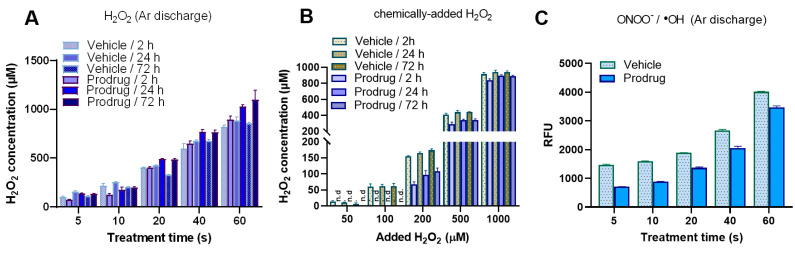
**Cold-plasma-derived ROS/RNS.** (**A**,**B**) H_2_O_2_ deposition and consumption kinetics after argon plasma treatment (**A**) and consumption of chemically added H_2_O_2_ (**B**); (**C**) deposition of ONOO^−^/^•^OH after argon plasma treatment. RFU = relative fluorescence units; Ar = argon; H_2_ = hydrogen; O_2_ = oxygen; n.d. = not determined.

**Figure A3:**
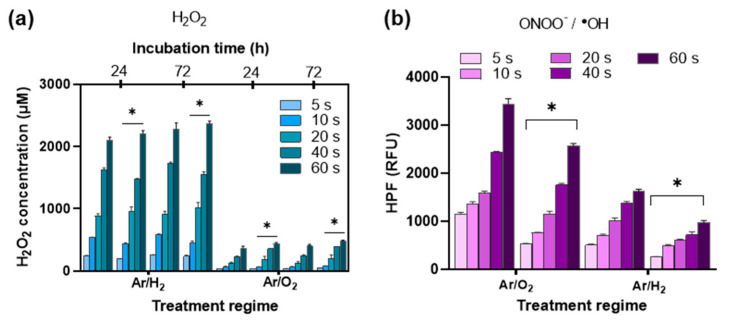
**Cold-plasma-derived ROS/RNS.** Quantification of cold-plasma-induced H_2_O_2_ (**a**) and ONOO^−^ (**b**) under Ar/H_2_ and Ar/O_2_ discharge regimes. (*) Prodrug-treated solution; Ar = argon; H_2_ = hydrogen; O_2_ = oxygen.

The applied plasma source generated short- and long-lived reactive species in the plasma gas phase and plasma–liquid interphase that penetrate the liquid bulk and potentially react with any (bio-) molecules, depending on their electric structure and reactivity [42,43]. H_2_O_2_ is a secondary product of hydroxyl radical (^•^OH) recombination in the gas phase, the gas–liquid interphase, and the main long-lived reactive species [44]. A strong deposition depending on the treatment duration and gas phase composition was observed in the plasma-treated prodrug solution and the plasma-treated vehicle (MeOH–H_2_O (1:1, *v*/*v*)) (Figure 2A and Figure A3). Relatively high concentrations of H_2_O_2_ were deposited by treating the sample under pure argon discharge regimes in an exposure-time-dependent manner (Figure 2A). At shorter cold plasma treatment times, the presence of the prodrug yielded lower deposition rates of H_2_O_2_ compared to the vehicle, an effect that leveled off for longer treatment times. The prodrug treatment with chemically added H_2_O_2_ showed similar behavior (Figure 2B), leveling off at over 200 μM. It can be assumed that this is due to the release of the parent drug. By contrast, when increasing the cold plasma treatment time to over 20 s, it seems that the produced reactive species mainly led to the deactivation (oxidation) of fenretinide and caused the slight production of H_2_O_2_ in the solution. Hydroperoxyl radicals (^•^OOH) and ^•^OH may be formed via the oxidation of the polyene double bonds of FenD or the pro-oxidation of the corresponding oxidized products upon prolonged plasma treatment that convert to H_2_O_2_ in an acidic pH medium [45,46]. Notably, allylic aldehydes derived from the oxidation of double bonds may contribute to damage to cancer cells and induce apoptosis [47,48]. Moreover, H_2_O_2_ concentrations in Ar/H_2_-treated samples were higher than in Ar and Ar/O_2_ discharge regimes (Figure A3(a)). The maximum H_2_O_2_ concentration was about 500 μM after 10 s cold plasma treatment under the Ar/H_2_ regime in vehicle samples, which was higher than the H_2_O_2_ concentration with Ar discharge in the same condition (~250 μM). The maximum H_2_O_2_ concentration was decreased with a 10 s plasma treatment time in the prodrug sample (Figure 2A and Figure A3(a)). The above observation indicates that the boronate moiety of the prodrug FenH was dissociated upon cold-plasma-induced H_2_O_2,_ most predominantly under Ar and Ar/H_2_ discharge regimes. Therefore, H_2_O_2_ played a central role in the oxidation of low-polarity boron–carbon bonds and aryl bond migration to release the cytotoxic drug. In addition, the concentration of chemically added H_2_O_2_ to the prodrug or vehicle was monitored, and the H_2_O_2_ concentration decreased in the presence of prodrugs, especially 24 h later (Figure 2B). Yet, H_2_O_2_ consumption was higher when H_2_O_2_ was chemically added (200 µM; Figure 2B) compared to 10 s cold plasma treatment (Ar: 80 µM; Ar/H_2_: 100 µM; Figure 2A and Figure A3(a)) at 24 h. The above observation proved our hypothesis about the central role of H_2_O_2_ in oxidizing the boron–carbon bond of the prodrug and the partial contribution of other reactive species, such as ONOO^−^. The slow decay of H_2_O_2_ is attributed to its slow in situ reaction with the prodrug, DMSO from the stock solution (0.1%), and released boric acid (perborate formation) [11,49].

Cold plasma treatment leads to the deposition of peroxynitrite (ONOO^−^) as the product of the reaction between superoxide radical (^•^O_2_^−^) and nitric oxide (NO). ONOO^−^ was quantified in liquid according to the HPF fluorescent assay [11,50]. Higher ONOO^−^ levels were observed in Ar (Figure 2C) compared to Ar/H_2_ and Ar/O_2_ regimes (Figure A3(b)). This is despite the fact that ^•^OH could interrupt ONOO^−^ quantification. However, the reduction in cold-plasma-induced ONOO^−^ in prodrug samples compared to vehicle samples could be attributed to the role of ONOO^−^ in prodrug activation in parallel with cold-plasma-induced H_2_O_2_ in the same treatment conditions. Therefore, H_2_O_2_ or ONOO^−^ may compete to activate the boronate ester prodrug and release the parent drug. Although the impact of ONOO^−^ can be expected to be less pronounced in kINPen jet liquid chemistry [51,52], its contribution in certain conditions to the activation of the prodrug (via boron–carbon bond oxidation) can be considered. In this study, the kINPen with Ar discharge was therefore chosen for the next experiments not only due to its known inhibitory activity against epithelial cancer cells [53,54] but also because it can produce relatively high amounts of H_2_O_2_ and ONOO^−^ only after 10 to 20 s plasma treatment, along with the slightest cold-plasma-induced fenretinide oxidation.

### 3.3. Prodrug Activation and In Situ Drug Release

**Figure 3:**
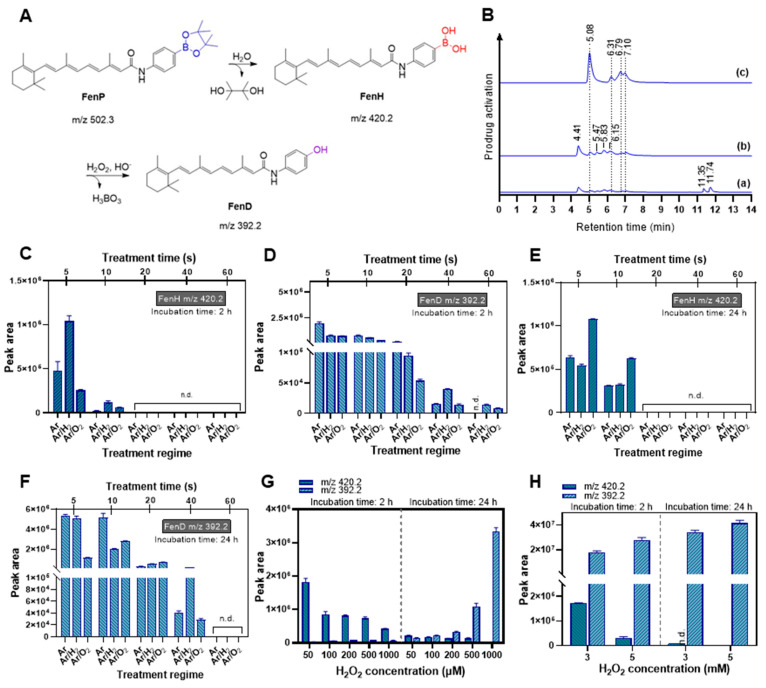
(**A**) Proposed activation mechanism of the prodrug upon cold plasma treatment and chemically added H_2_O_2_; (**B**) HPLC–MS^2^ of the boronate pinacol ester fenretinide prodrug solution incubated at ambient temperature for 2 h ((**a**), FenP) and 72 h ((**b**), hydrolysis step: FenH in solution) and after plasma treatment ((**c**), FenD release); (**C**–**F**) abundance (combined sum of peak areas) of FenH and FenD after cold plasma treatment with varying admixture gases (Ar, Ar/H_2_, and Ar/O_2_ regimes) at 2 h (FenH (**C**) and FenD (**D**)) and 24 h (FenH (**E**) and FenD (**F**)); (**G**,**H**) abundance of FenH/FenD after incubation with chemically added H_2_O_2_ at concentrations up to 1000 μM (**G**) and 5 mM (**H**) for specific durations. Ar = argon; H_2_ = hydrogen; O_2_ = oxygen; n.d. = not determined.

RONS-responsive prodrugs can be activated depending on the chemical structure of the boronate group, the concentration and reactivity of RONS, the pH, and the presence of other (bio-) molecules or antioxidants in the cell culture medium or solution [8,10]. Biologically inactive prodrugs can be converted into cytotoxic drugs upon RONS generation by metabolic processes in the tumor microenvironment through “triggered release” mechanisms. The activation of the fenretinide prodrug (FenP) by cold plasma is mechanistically outlined in Figure 3A. The initial boronate pinacol ester is first hydrolyzed, accompanied by the release of pinacol and the slow formation of fenretinide aryl boronic acid (FenH) for 72 h at ambient temperature. The relatively slow hydrolysis rate is attributed to the strong coordination of phenyl to an amide group [55]. Achilli et al. found that the boronate ester cleavage kinetics depends on the substituents on the phenolic aromatic ring and the pH [55]. The boron atom of FenH has an empty *p*-orbital and can thus be attacked by RONS (predominantly H_2_O_2_), resulting in a phenol-O-B(OH)_2_ intermediate via 1,2 rearrangement, yielding a phenyl borate. Subsequently, phenyl borate is hydrolyzed to the corresponding phenolic parent drug (FenD) and boric acid (H_3_BO_3_).

**Figure A4:**
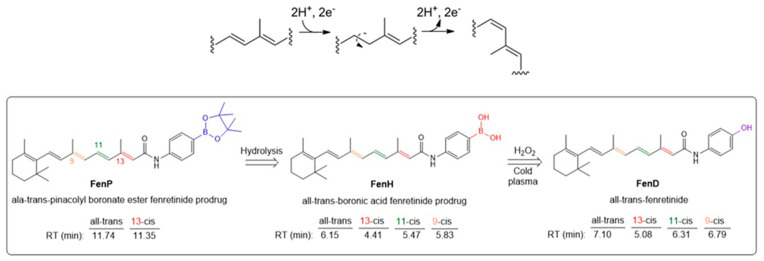
Plausible mechanism of fenretinide isomerization [56].

The proposed activation process upon cold-plasma-induced RONS or the direct addition of H_2_O_2_ was monitored in the solution using ESI-MS(+) and then HPLC-MS^2^. Therefore, the efficiency of various cold plasma treatment feed gas regimes, including Ar, Ar/H_2_, and Ar/O_2_ discharges, in activating the fenretinide prodrug was examined. The first screening prior to treatment led to the disappearance of FenP MS signals at *m/z* 502.3 (retention time (t_R_) in chromatogram: 11.74 and 11.35 min) and the appearance of a new set of signals at *m/z* 420.2 and 392.2 (Figure 3B(a); set of peaks at t_R_ 4.41 to 7.10 min). The t_R_ of FenP at 11.74 and 11.35 min might relate to the trans isomer and 13-cis isomer, respectively (Figure 3B(a)). This is attributed to the bulky pinacol ester pro-moiety in FenP that does not undergo further isomerization compared to FenH and FenD. Treating the FenH solution with cold plasma led to the observation of a new set of peaks in the chromatogram at t_R_ 5.08, 6.31, 6.79, and 7.10 min, belonging to FenD, in all cold-plasma-treated solutions (Figure 3B(c)). Peaks at t_R_ 4.41, 5.47, 5.83, and 6.15 min in the chromatogram of the FenH-treated solution are attributed to the 13-cis, 11-cis, 9-cis, and trans isomers, respectively (Figure 3B(b) and Figure A4), which is in accordance with the literature regarding the ratio of geometric isomers of retinal in equilibrated methanolic solutions [56]. The determination of the geometric isomeric composition and the corresponding t_R_ peaks of retinoic acid was also reported utilizing HPLC-MS^2^ [56,57]. In this context, the known retinal isomers’ composition ratio is as follows: trans > 13-cis > 9-cis > 11-cis [56]. The disappearance of four distinct isomeric peaks of FenH in the chromatogram and the appearance of four new peaks belonging to FenD confirm the activation of the fenretinide prodrug upon cold-plasma-induced RONS.

**Figure A5:**
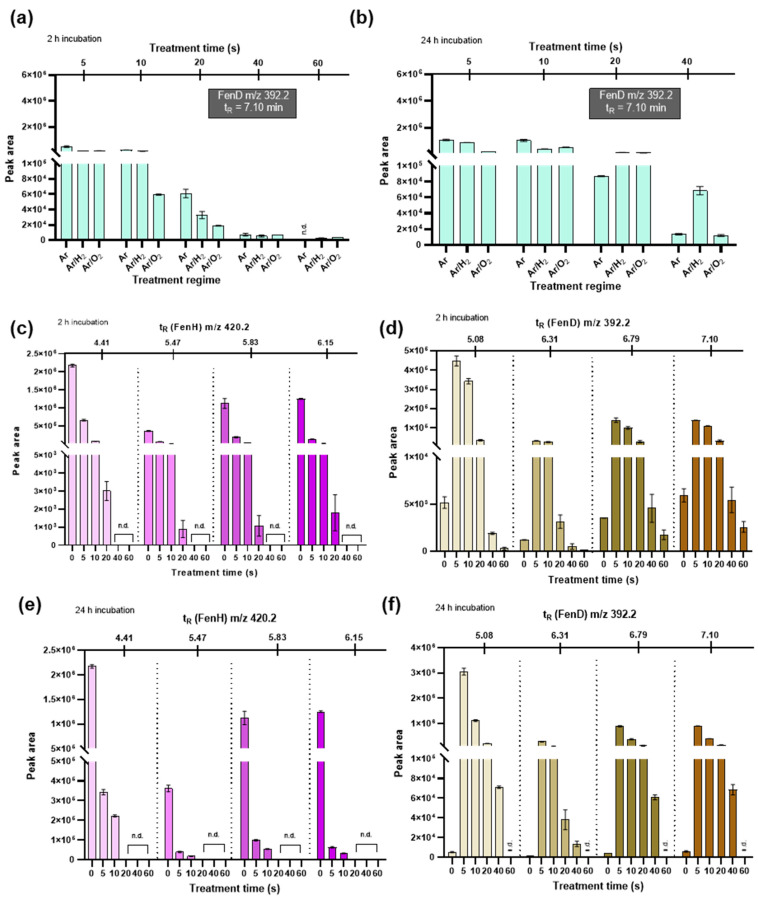
Trans-FenD release at t_R_ = 7.10 min in chromatogram upon plasma treatment incubated for 2 h (**a**) and 24 h (**b**); abundance (peak area) of FenH degradation at *m/z* 420.2 and FenD release at *m/z* 392.2 upon plasma treatment under the Ar/H_2_ discharge regime at 2 h (**c**,**d**) and 24 h (**e**,**f**) incubation time. Ar = argon; H_2_ = hydrogen; O_2_ = oxygen; n.d. not determined.

**Figure A6:**
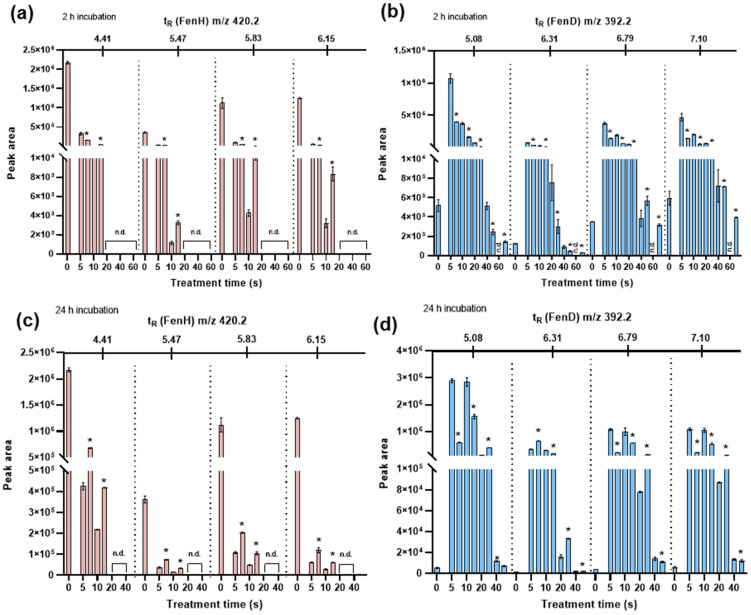
The abundance (peak area) of FenH degradation at *m/z* 420.2 and FenD release at *m/z* 392.2 upon cold plasma treatment under Ar and Ar/O_2_ discharge regimes with 2 h (**a**,**b**) and 24 h (**c**,**d**) incubation. (*) Ar/O_2_ discharge regime; Ar = argon; H_2_ = hydrogen; O_2_ = oxygen; n.d. = not determined.

The screening of four FenH isomers revealed the complete disappearance of FenH within 15–20 s of plasma treatment in all discharge regimes (Figure 3C). Plasma treatment efficiently triggered the rearrangement of the aromatic ring, yielding the loss of the boronate group and the release of FenD (Figure 3D). Prolonged incubation of the cold-plasma-treated solutions for 24 h displayed no further degradation (Figure 3E). The FenD release remained stable for 10–20 s treatment and decreased significantly when increasing the treatment time to 60 s (Figure 3D). After prolonged incubation, the abundance of FenD increased about 8-fold for 5–10 s plasma treatment (Ar discharge regime). Under the influence of the Ar/O_2_ plasma, the abundance of FenD after prolonged incubation decreased, particularly for longer treatment durations (Figure 3D,F). However, the complete release of FenD was observed after 10 s plasma treatment. Results showing the same trend were also observed by only monitoring the trans-FenD isomer at t_R_ 7.10 min in the HPLC chromatogram (Figure A5(a,b)). The abundance of the trans-FenH isomer at t_R_ 6.15 min was decreased when increasing the treatment time to 20 s. Moreover, the abundance of cis/trans isomers before and after activation by cold-plasma-induced RONS changed (Figure A5(c–f) and Figure A6(a–d)).

However, drug oxidation can be assumed by increasing the treatment time to over 20 s in all gas compositions. The oxidation of unsaturated polyene double bonds under the Ar/O_2_ regime is inevitable, resulting in the reduction in FenD abundance upon prolonging the treatment duration. ROS can oxidize double bonds to allylic peroxides, allylic aldehyde, and epoxides, which can be further oxidized to form complementary products [46,58]. Cold-plasma-generated short-life ROS, such as ^•^OH generated in liquid, may lead to the chemical decomposition of double-bond chains via proton abstraction from allylic or benzylic positions and direct electron abstraction/addition from/to the π resonance system [46]. Hence, increasing the treatment duration causes more exposure of the prodrug in solution to short-life ROS, consequently enhancing the likelihood of oxidation reactions. By comparing the abundance of FenD and FenH in solution (Figure 3C–F), the Ar discharge and admixed Ar/H_2_ discharge regimes displayed slightly more substantial prodrug degradation and, ultimately, higher FenD abundance in solution than the Ar/O_2_ regime. This is despite the fact that the deposited H_2_O_2_ concentration in the Ar/H_2_ discharge regime was higher than in the Ar discharge regime. As expected, this phenomenon suggests that the activation process of the prodrug by plasma may not depend solely on cold-plasma-deposited H_2_O_2_ but also on other reactive species, such as ONOO^−^, which can also promote the boron oxidation reaction [59]. The deposited ONOO^−^ in the argon-driven kINPen jet [51,52] may promote the activation of RONS-responsive prodrugs along with cold-plasma-deposited H_2_O_2_ [11,52,60].

**Figure A7:**
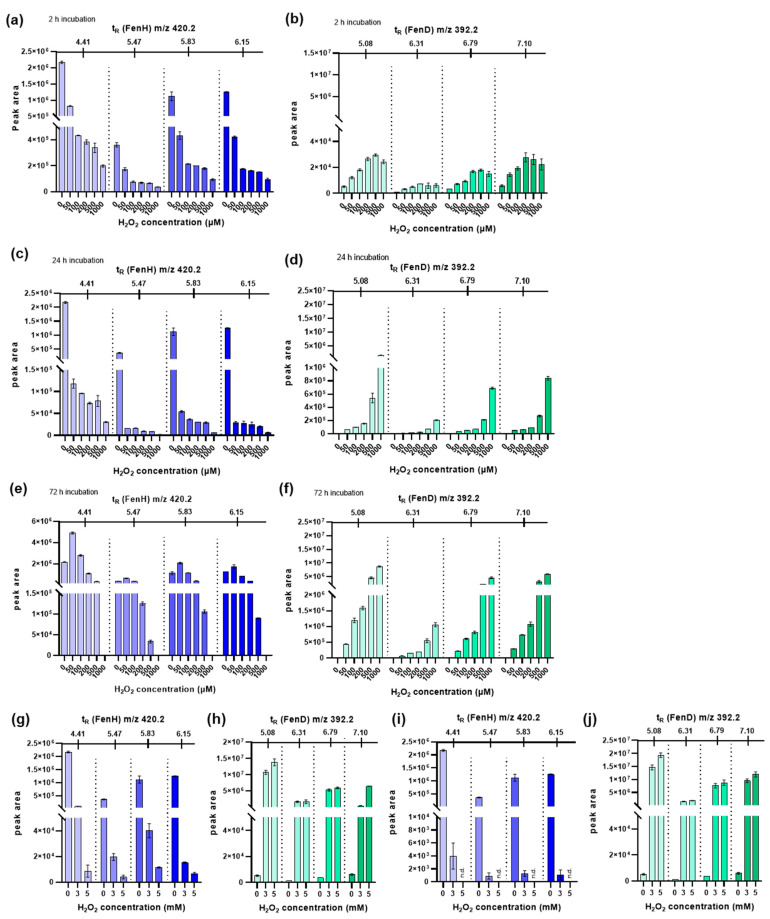
The abundance (peak area) of FenH degradation at *m/z* 420.2 and FenD release at *m/z* 392.2 upon addition of 50 µM to 1000 µM H_2_O_2_ after 2 h (**a**,**b**), 24 h (**c**,**d**), and 72 h (**e**,**f**) of incubation; 3 mM and 5 mM H_2_O_2_ after 2 h (**g**,**h**), and 24 h (**i**,**j**) of incubation. n.d. = not determined.

In parallel, the proposed activation mechanism was also studied with chemically added H_2_O_2_ to prodrug solution samples. The hydrolyzed aryl boronic acid (FenH) was not fully converted to FenD by 500 µM H_2_O_2_ within 24 h of incubation (Figure 3G–H). When doubling the H_2_O_2_ concentration, the abundance of FenD increased about 3-fold and further increased with increasing amounts of H_2_O_2_ and prolonged incubation times (up to 5 mM for 24 h). However, the abundance of FenD in samples treated with up to 1000 μM H_2_O_2_ remained below the obtained abundance value in plasma-treated samples. The 5-fold enhancement of FenD after plasma treatment (treatment time: 10 s; Ar discharge) was comparable to the addition of 250–500 μM H_2_O_2_. In comparison, decreasing H_2_O_2_ production and increasing the concentration of FenD in solution upon the plasma treatment of the prodrug for up to 20 s (Figure 3) confirmed not only successful prodrug activation but also our hypothesis regarding the role of other reactive species, such as ONOO^−^, in the activation mechanism triggered by cold plasma [61]. The peak areas obtained from HPLC-MS^2^ of trans/cis isomers of FenH and FenD prior to and after the addition of H_2_O_2_ are changed (Figure A7).

The high concentration of H_2_O_2_ causes the relatively fast oxidative cleavage of the boron–carbon bond in FenH after 2 h of incubation and the complete release of FenD. High concentrations of H_2_O_2_ can also be associated with cytotoxicity and tissue damage by producing ^•^OH [62]. Tumor microenvironment H_2_O_2_ concentrations range between 0.1 and 1.5 mM across cancer types and stages, which is higher than that in non-malignant, non-inflamed tissues [63,64]. Hence, the mild deposition of H_2_O_2_ by cold plasma could mimic the tumor microenvironment in a time- and space-resolved manner for cancer treatment.

**Figure 4:**
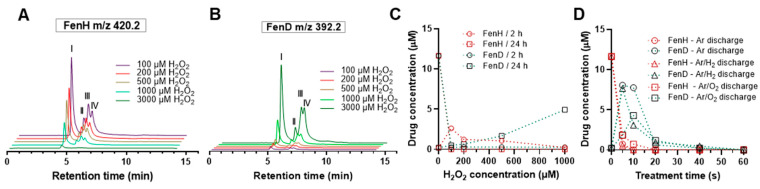
Prodrug cleavage and fenritinide release upon H_2_O_2_ addition. FenH degradation (**A**) and FenD release (**B**) upon incubation with different concentrations of H_2_O_2_ after 24h of incubation. I: 13-cis; II: 11-cis; III: 9-cis; IV: trans. Quantitative FenH degradation and FenD release in solution upon direct addition of H_2_O_2_ after 24h of incubation (**C**) and plasma treatment (**D**) (Ar, Ar/O_2,_ and Ar/H_2_ discharge regime) at a given incubation time points. Ar = argon; H_2_ = hydrogen; O_2_ = oxygen.

**Figure A8:**
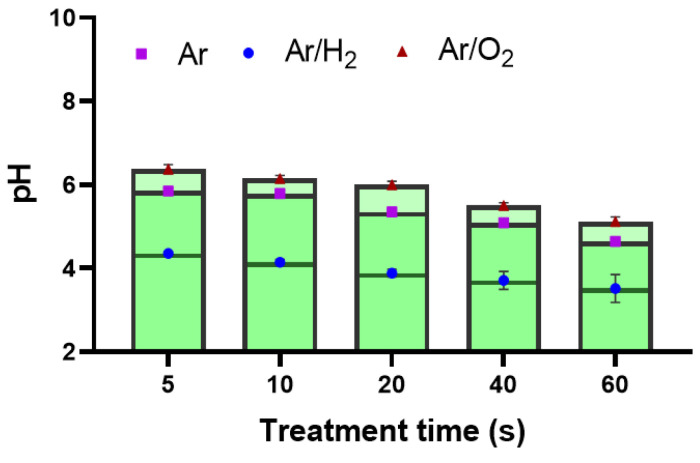
pH of solution (mixture of H_2_O–MeOH (1:1, *v*/*v*)) after plasma treatment ignited with varying gas admixtures.

The reduction in isomeric peaks of FenH at *m/z* 420.2 (Figure 4A) and the simultaneous increase in isomeric peaks of FenD as the parent drug (Figure 4B) at *m/z* 392.2 confirmed the activation of the boronate prodrug in a H_2_O_2_-concentration-dependent manner. When increasing the H_2_O_2_ concentration, FenH was fully degraded, and FenD release was relatively slow and increased with a longer incubation time of 24 h (Figure 4C). In contrast, after 10 s plasma treatment using Ar discharge, FenD release significantly increased, while almost all FenH was degraded at this time (Figure 4D). This phenomenon revealed that activation upon treatment with H_2_O_2_ (up to 500 μM) is slower than in the plasma treatment (Ar discharge). When prolonging the incubation time (24 h), adding 1000 μM H_2_O_2_ showed a better performance in releasing the parent drug (less than half of the FenD was released). Notably, complete FenD release was observed when increasing the H_2_O_2_ concentration (up to 3 mM samples incubated for 24 h) or using longer incubation times (up to 72 h for samples containing 1000 μM) (Figure A7(e–j)). This observation suggests that the boronate ester bond in the prodrug is stable enough to resist spontaneous cleavage under normal physiological conditions but can be cleaved effectively in the presence of H_2_O_2_ at higher concentrations or with longer incubation times. In addition, the produced RONS contribute to a change in the pH of the solution, especially hydroperoxyl radicals (HO_2_) and (H)N_x_O_y_ species. The pH dropped significantly with increasing cold plasma treatment time, associated with the significant production of HO_2_ and cold-plasma-derived RNS-based acids (Figure A8). The pH of the solution after plasma treatment with pure argon as the feed gas or when argon was admixed with H_2_ was more acidic than in the Ar/O_2_ case, reflecting the varying formation of nitric acid, nitrous acid, and H_2_O_2_ [18,65]. The pH value in the chemically added H_2_O_2_ treatment conditions is more related to a mildly acidic environment [11]; however, a relatively high amount of H_2_O_2_ is needed to fully activate the prodrug. In contrast, after 10–20 s plasma treatment, complete prodrug activation was observed in an acidic plasma-treated medium (Figure 3). It could be proposed that the boronate ester FenP is hydrolyzed to form aryl boronic acid FenH under slightly acidic conditions, which was further oxidized to the phenolic compound FenD, triggered by cold-plasma-induced H_2_O_2_ and ONOO^−^ through the oxidation of boron–carbon bonds, aryl bond migration, and the loss of boric acid [11]. Comparing the maximum FenD release after 10–20 s plasma treatment (Ar discharge) with the direct addition of H_2_O_2_ emphasizes the role of cold-plasma-induced ONOO^−^ in oxidizing the boron–carbon bond in the prodrug [11,59]. The reduction in parent drug release with increasing treatment time (over 20 s) indicates the sensitivity of FenD (unsaturated double bonds) to oxidation by cold-plasma-induced ROS such as atomic oxygen (^•^O), ^•^OH, and singlet oxygen (^1^O_2_) produced specifically in the Ar/O_2_ discharge regime. Therefore, the activation of the prodrug with ROS-sensitive structures may have limited stability in harsh cold-plasma-derived reactive species mixtures.

**Figure A9:**
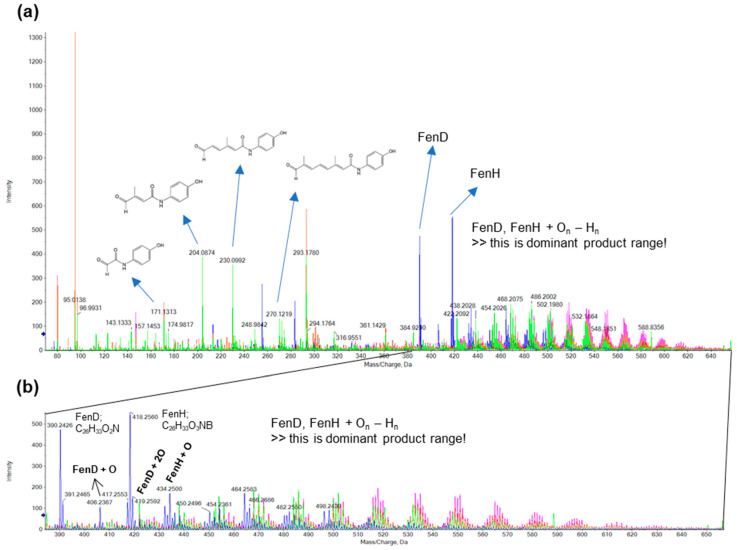
(**a**) ESI-MS(−) of samples before and after cold plasma treatment under Ar (pink color), Ar/H_2_ (red color), and Ar/O_2_ (green color) regimes; (**b**) expanded spectrum from *m/z* 390 to 650.

The oxidative degradation of FenD was monitored using high-resolution ESI-MS(–) (Figure A9). Polyene unsaturated double bonds underwent reactions in the presence of ^•^O and ^•^OH (hydroxylation and dihydroxylation), ^•^OOH and O_2_ (epoxidation), and ^1^O_2_ (to form allylic hydroperoxides) [19,61,66]. Both FenH (*m/z* 418.25) and FenD (*m/z* 390.24) were oxidized with longer plasma treatment (>20 s). Especially in the case of plasma treatment with Ar and Ar/H_2_ discharges, the addition of one or more oxygen atoms, combined with the loss of hydrogen atoms, yielded a series of products [FenH + O_n_ − H_n_] [FenD + O_n_ − H_n_] above *m/z* 424 (Figure A9). Molecular fragmentation was detected upon the impact of the Ar/O_2_ plasma discharge on FenD and FenH, showing the main signals at *m/z* 164.06, 204.08, 230.09, and 270.12. Characteristic 40 Da and 26 Da mass differences can be observed as intervals, with the 40 Da mass indicating the loss of C_2_H_2_O, while the 26 Da mass indicates the replacement of a propene group (C_3_H_6_; 42 Da *m/z*) by oxygen (16 Da *m/z*) [67]. A signal at *m/z* 293.17 is observed before and after cold plasma treatment, attributed to the fragmentation of FenD. However, it is unclear whether oxidation products affect the biological activity of FenD or whether they are even toxic to cancer cells. Due to the complex RONS induction by cold plasma, further experiments are needed to identify potential synergistic effects of cold-plasma-induced RONS and RONS-responsive prodrugs and chemotherapeutic agents or their oxidized products [68,69,70].

### 3.4. Combined Cold Plasma and FenP or FenD Treatment Showed Additive Cytotoxic Effects

**Figure 5:**
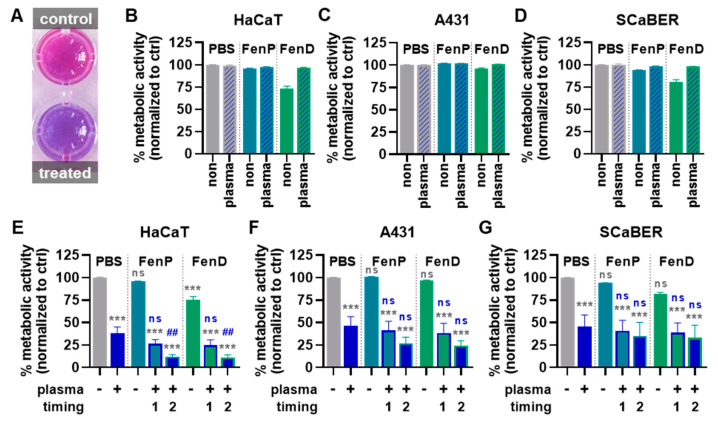
**Metabolic activity reduction in three epithelial cell lines with plasma and drug combination treatments**. (**A**) Representative macroscopic image of the resazurin assay; (**B**–**D**) metabolic activity of HaCaT (**B**), A431 (**C**), and SCaBER (**D**) cells 72 h after cold plasma treatment of PBS or the addition of non-treated and plasma-treated FenP or FenD; (**E**–**G**) metabolic activity of HaCaT (**E**), A431 (**F**), and SCaBER (**G**) cells 72 h after cold plasma treatment of the cells alone or prior (timing 1) or after (timing 2) addition of FenP or FenD. Data are mean and S.E. of three independent experiments. Statistical analysis (**E**–**G**) was performed using one-way analysis of variances (ANOVA) with Dunnett’s post hoc test against untreated PBS vehicle (gray symbols) or cells treated with plasma alone (blue symbols). *** = *p* < 0.001 compared to PBS without plasma exposure (-); ## = *p* < 0.01 compared against PBS with plasma exposure (+); ns = *p* > 0.05.

**Figure 6:**
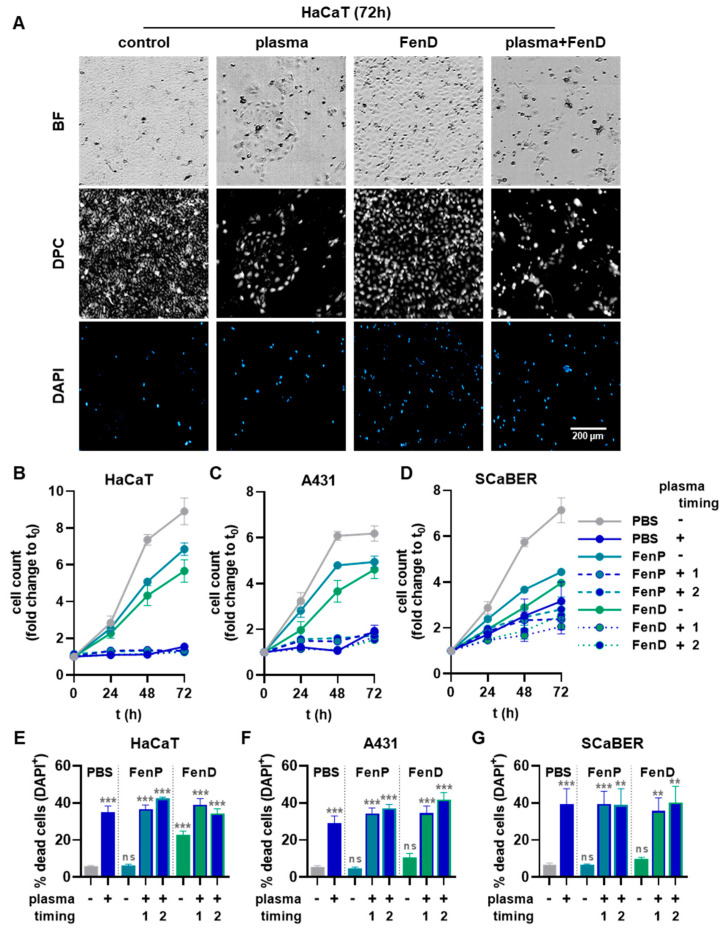
**Viability in three epithelial cell lines with plasma and drug combination treatments**. (**A**) Representative brightfield (BF, (**top**)), digital phase contrast (DPC, (**middle**)), and DAPI (**bottom**) microscopy of HaCaT cells 72 h following plasma, drug, and plasma–drug combination treatments; (**B**–**D**) cell count (growth) of HaCaT (**B**), A431 (**C**), and SCaBER (**D**) cells up to 72 h after cold plasma treatment of PBS or the addition of non-treated and plasma-treated FenP or FenD; (**E**–**G**) number of dead (DAPI^+^) cells in HaCaT (**E**), A431 (**F**), and SCaBER (**G**) cells 72 h after cold plasma treatment of the cells alone or prior (timing 1) or after (timing 2) addition of FenP or FenD. Data are mean and S.E. of three independent experiments. Statistical analysis (**E**–**G**) was performed using one-way analysis of variances (ANOVA) with Dunnett’s post hoc test against untreated PBS vehicle (gray symbols). ** = *p* < 0.01; *** = *p* < 0.001; ns = *p* > 0.05.

To investigate the effects of plasma-treated drugs in a pilot experiment, the three cell lines were exposed to untreated FenP or FenD or to the drugs treated with plasma for 15 s prior to cell exposure. After 72 h, the resazurin assay (Figure 5A) showed slight reductions in metabolic activity for the FenP treatment and comparably increased reductions in metabolic activity for the FenD treatment, which was abrogated by drug pre-treatment with plasma (Figure 5B–D). For further cell experiments, only untreated FenP and FenD were used and combined with the direct cold plasma treatment of the epithelial cells. In HaCaT cells, metabolic activity was significantly decreased upon plasma treatment, and combination treatments increased this effect. The treatment of HaCaTs with plasma after the addition of the drugs (timing 2) reduced the metabolic activity even more than the treatment with plasma prior to drug addition (timing 1), and it differed significantly from the single (mono) treatment with plasma (Figure 5E). A comparable but less pronounced reduction in metabolic activity was also found in A431 (Figure 5F) and SCaBER cells (Figure 5G). Generally, the FenD treatments reduced the metabolic activity to a greater extent than FenP treatments at the same concentrations in all three cell lines. Live-cell imaging was performed immediately before (0 h) as well as 24 h, 48 h, and 72 h after treatment (Figure 6A). Algorithm-based quantitative image analysis was performed to segment cells and calculate their cell growth. In parallel, the analysis of DAPI^+^ yielded the number of dead cells. The increase in cell count over time was reduced by FenP and FenD in all cell lines. In HaCaT (Figure 6B) and A431 cells (Figure 6C), plasma and combination treatments almost completely inhibited cell growth for up to 72 h, and in SCaBER cells (Figure 6D), a strong reduction in cell growth was visible in plasma and combination treatments. The detection of dead cells showed the significant cytotoxic effects of plasma and combination treatments in all three cell lines (Figure 6E–G).

For the identification of synergistic effects, the coefficient of drug interaction (CDI) for cold plasma and drug combination treatments was calculated based on the resazurin assay results (Table 1). The combination of cold plasma and FenP or FenD showed additive effects (CDI < 1) in all three cell lines investigated, except for the cold plasma and FenD combination (timing 1) in SCaBER cells. The lowest CDIs were found in HaCaT cells, indicating the greatest additive effect in this cell line. The HaCaT cell line is an immortalized keratinocyte line that is regarded as non-malignant since it is not a cancer cell line. Still, it is highly proliferative and, therefore, a common model for proliferative skin diseases (e.g., psoriasis) [71,72]. This work focuses on showing the concept of prodrug activation but not on verifying the selectivity of fenretinide toward cancer cells. In all cell lines tested here, the additive effect was more pronounced when the drug was added prior to plasma treatment (timing 2) compared to its addition following plasma exposure (timing 1). In HaCaT and SCaBER cells, the additive effect of FenP and plasma was stronger than that of FenD and plasma (CDI_FenP ½_ < CDI_FenD 1/2_), while in A431, it was relatively similar for both drugs.

## 4. Conclusions

The feasibility of utilizing the pro-oxidant activity of cold physical plasma for the time- and space-controlled release of anticancer drugs was successfully tested. A boronate pinacol ester fenretinide prodrug was activated by plasma-generated RONS via the oxidative cleavage of a boron–carbon bond, followed by the release of free fenretinide. The efficacy of the release process was dependent on the discharge parameter of the cold plasma, especially the feed gas composition settings that govern the RONS output. In good accordance with the proposed reaction mechanism, H_2_O_2_ and ONOO^−^ were found to be mainly responsible for the drug release. Prolonged cold plasma treatment durations conferred oxidative damage through short-lived reactive species, mainly by radical-driven addition reactions at the molecule’s unsaturated terpene chain. When combined in vitro, an additive effect of the prodrug and cold plasma treatment in three different epithelial cell lines corroborated the analytical findings. The results emphasize the opportunity to minimize undesired side effects by locally activating an anticancer agent by cold-plasma-induced RONS and increase its efficacy by leveraging the direct impact of plasma-generated RONS. Future work to substantialize the concept includes a screening of candidate drugs and more complex (in vivo) models.

## Figures and Tables

**Table 1:** Coefficients of drug interactions (CDIs) in all three cell lines and treatments investigated.

Treatment			HaCaT		A431		SCaBER	
Drug	Plasma	Timing	Metabolic Activity	CDI	Metabolic Activity	CDI	Metabolic Activity	CDI
-	+		0.38	-	0.46	-	0.46	-
FenP	-		0.96	-	1.01	-	0.94	-
FenP	+	1	0.26	0.73	0.41	0.88	0.41	0.95
FenP	+	2	0.12	0.33	0.27	0.57	0.35	0.82
FenD	-		0.75	-	0.96	-	0.82	-
FenD	+	1	0.24	0.86	0.38	0.86	0.39	1.04
FenD	+	2	0.11	0.40	0.24	0.54	0.33	0.88

## Data Availability

The underlying data of this work are available from the corresponding authors upon reasonable request.
